# Evaluation of Corneal Densitometry in Patients with Type 2 Diabetes Mellitus

**DOI:** 10.3390/medicina62101887

**Published:** 2026-09-29

**Authors:** Armin Kasumović, Ines Matoc Kasumović, Aida Kasumović Bećirević, Ivan Merdžo, Ajla Jahić, Marija Veselinović, Mirko Jankov, Ivan Sabol, Sanja Sefić Kasumović

**Affiliations:** 1 Department of Ophthalmology, Private Eye Clinic Dr. Sefić, 71000 Sarajevo, Bosnia and Herzegovina; kasumovic.armin@gmail.com (A.K.); inesmatoc@gmail.com (I.M.K.); ajla.jahic@icloud.com (A.J.); info@sefic.ba (S.S.K.); 2 Department of Ophthalmology, University Hospital Mostar, University of Mostar School of Medicine, 88000 Mostar, Bosnia and Herzegovina; ivan.merdzo@mef.sum.ba; 3 Department of Ophthalmology, University Clinical Center Niš, 18000 Niš, Serbia; drmarija.veselinovic@gmail.com; 4 Centre for Microsurgery LaserFocus, 11000 Belgrade, Serbia; mirkojankov@mac.com; 5 Institut für Refraktive und Ophthalmo-Chirurgie, 8002 Zurich, Switzerland; 6 Division of Molecular Medicine, Ruđer Bošković Institute, Bijenička Cesta 54, 10000 Zagreb, Croatia; ivan.sabol@irb.hr

**Keywords:** diabetes mellitus type 2, corneal densitometry, scheimpflug tomography, corneal transparency, diabetic keratopathy

## Abstract

*Background and Objectives*: Diabetes mellitus (DM) may induce structural and biochemical changes in the cornea that can potentially affect corneal transparency. This study aimed to compare corneal optical density (COD) between patients with type 2 diabetes mellitus (T2DM) and healthy controls and to evaluate its association with disease duration. *Materials and Methods*: This observational cross-sectional study included 90 eyes of 90 participants, with only one eye per participant included in the analysis. The study population comprised 45 eyes from patients with T2DM and 45 age- and gender-matched healthy control eyes. The study was conducted at a specialized ophthalmology center between September 2025 and April 2026. All participants underwent a comprehensive ophthalmic examination followed by corneal densitometry and pachymetry assessment using the Pentacam AXL (Oculus Optikgeräte GmbH, Wetzlar, Germany). COD was analyzed across the total, anterior, central, and posterior corneal layers within the 0–2 mm, 2–6 mm, and 6–10 mm annular zones. COD values and central corneal thickness (CCT) were compared between groups, and correlations between disease duration and COD parameters were evaluated. *Results*: No statistically significant differences in COD were observed between patients with T2DM and healthy controls across any of the analyzed corneal layers or annular zones (all *p* > 0.05). CCT also did not differ significantly between groups (*p* = 0.817). No significant correlations were identified between disease duration and COD parameters (all *p* > 0.05). *Conclusions*: In this age- and gender-matched cohort, patients with T2DM did not demonstrate statistically significant differences in corneal densitometry or CCT compared with healthy controls. Disease duration was not significantly associated with COD, suggesting that other factors, including long-term glycaemic control and disease severity, may contribute to diabetes-related corneal alterations.

## 1. Introduction

The global prevalence of diabetes mellitus (DM) continues to increase at an alarming rate, making it one of the greatest public health challenges of the 21st century. According to the current data, more than 537 million adults are currently living with DM, and this number is projected to approach 700 million by 2045. Persistent hyperglycaemia induces a wide range of metabolic disturbances that progressively damage both the microvasculature and macrovasculature, resulting in substantial morbidity, premature mortality, and an increased socioeconomic burden [1,2,3].

Although diabetic retinopathy remains the most recognized ocular manifestation of DM, increasing evidence indicates that diabetes also induces structural and functional alterations of the cornea, collectively referred to as diabetic keratopathy [4,5]. These corneal abnormalities may precede clinically evident ocular complications and can negatively influence wound healing, refractive surgery outcomes, and overall corneal health [5,6]. Persistent hyperglycemia promotes the accumulation of advanced glycation end products (AGEs), oxidative stress, chronic inflammation, and endothelial dysfunction, leading to alterations in the corneal extracellular matrix and collagen architecture [5,6]. These diabetes-related changes have been associated with impaired epithelial regeneration, reduced corneal sensitivity, endothelial dysfunction, stromal edema, and decreased corneal transparency [5]. Since many of these alterations are subtle and may not be detected during routine slit-lamp examination, objective imaging techniques capable of identifying early microstructural corneal changes have gained increasing clinical importance [7,8].

The Pentacam (Oculus, Wetzlar, Germany), a Scheimpflug imaging-based corneal tomography system, is one of the most widely used instruments for anterior segment evaluation. In addition to providing objective, non-contact assessment of corneal topography and pachymetry, it also incorporates a Scheimpflug-based corneal densitometry module, enabling quantitative assessment of corneal transparency [9,10]. Corneal densitometry quantifies backward light scatter and serves as an objective indicator of corneal optical density (COD), expressed in grayscale units (GSU). Because corneal transparency depends on the highly organized arrangement of collagen fibrils and maintenance of stromal hydration, increased densitometry values may reflect subtle structural alterations that are not clinically apparent [7,11].

Previous studies have reported conflicting results regarding corneal densitometry in DM. Gao et al., Özyol et al., Elagamy et al., Coşkun et al., and Calvo-Maroto et al. reported increased corneal densitometry in diabetic patients, whereas Ramm et al. observed lower densitometry values [12,13,14,15,16,17]. These discrepancies may be explained by differences in study populations, diabetes duration, glycaemic control, presence of diabetic retinopathy, imaging protocols, and methodological heterogeneity. Furthermore, relatively few studies have examined whether disease duration itself is associated with corneal densitometric changes, and the available evidence remains limited. Therefore, further investigation is warranted to better characterize diabetes-associated corneal alterations using objective imaging techniques.

The present study aimed to compare corneal densitometry between healthy individuals and patients with type 2 diabetes mellitus (T2DM) using Pentacam AXL and to evaluate whether disease duration is associated with corneal densitometry parameters. We hypothesized that diabetic eyes would demonstrate increased COD compared with healthy controls, supporting the potential role of Pentacam AXL in detecting subtle diabetes-related corneal changes.

## 2. Materials and Methods

### 2.1. Study Design and Participants

This observational cross-sectional study was conducted between September 2025 and April 2026 at the Private Eye Clinic “Dr. Sefić”, Sarajevo, Bosnia and Herzegovina. The study included 90 eyes of 90 participants, comprising 45 eyes from patients with T2DM and 45 eyes from healthy controls. Only one eye per participant was included in the analysis. The right eye was selected when it fulfilled all eligibility criteria; if the right eye did not meet the eligibility criteria, the left eye was included instead. To minimize potential confounding by age and gender, participants were matched in a 1:1 ratio according to age and gender, with each participant with T2DM matched to a healthy control of the same age and gender. Matching was performed by an independent third person who had no access to corneal densitometry measurements or study outcomes. Eligible participants were between 50 and 70 years of age and underwent a comprehensive ophthalmic examination together with Scheimpflug corneal tomography using the Pentacam AXL (Oculus Optikgeräte GmbH, Wetzlar, Germany, software version 6.12r87). Pentacam measurements were performed between 10:00 AM and 4:00 PM, without systematic differences in measurement timing between groups, and under dimmed ambient light. The diagnosis of T2DM was established according to the participants’ medical history and ongoing antidiabetic treatment. Exclusion criteria included previous ocular surgery, corneal disease, glaucoma, ocular trauma, active ocular inflammation or infection, spherical or cylindrical refractive error exceeding ±3.00 diopters, contact lens wear, pregnancy, and systemic or ocular conditions known to affect corneal transparency independently of diabetes.

The study was conducted in accordance with the principles of the Declaration of Helsinki and was approved by the Ethics Committee of the Private Eye Clinic “Dr. Sefić” (Approval No. 2025-6/01; approval date: 6 June 2025). Written informed consent for participation in the study was obtained from all participants prior to study-related examinations and data collection.

### 2.2. Ophthalmic Examination

All participants underwent a comprehensive ophthalmic examination including measurement of best-corrected visual acuity (BCVA), slit-lamp biomicroscopy of the anterior segment, Goldmann applanation tonometry, dilated fundus examination, and optical coherence tomography when clinically indicated. Patients with diabetes were additionally evaluated for the presence and severity of diabetic retinopathy according to fundus examination findings.

### 2.3. Corneal Densitometry Assessment

Corneal densitometry measurements were obtained using the Pentacam AXL (Oculus Optikgeräte GmbH, Wetzlar, Germany; software version 6.12r87), a rotating Scheimpflug imaging system that provides objective quantification of corneal backscattered light expressed in standardized grayscale units (GSU), ranging from 0 (maximum transparency) to 100 (maximum opacity), with higher GSU values indicating greater corneal light scatter and reduced transparency [10,18,19]. All examinations were performed by the same trained medical technician experienced in Pentacam AXL image acquisition. The operator was masked to group allocation and was not informed whether the participant belonged to the T2DM or healthy control group. Several scans were acquired per eye when necessary to obtain an optimal-quality examination, and the single best scan fulfilling the manufacturer’s quality specification (“OK”) was selected for the final analysis. COD was automatically calculated for three concentric annular zones (0–2 mm, 2–6 mm, and 6–10 mm) and for four corneal layers (anterior, central, posterior, and total corneal thickness). Mean COD values for each region from the selected scan were exported for statistical analysis. Central corneal thickness (CCT) was also recorded. Although repeatability was not specifically assessed in the present cohort, previous studies have demonstrated high repeatability of Pentacam-based corneal densitometry measurements, with intraclass correlation coefficients exceeding 0.90 across corneal layers and annular zones [18,19].

### 2.4. Statistical Analysis

Statistical analysis was performed using IBM SPSS Statistics software version 32.0 (IBM Corp., Armonk, NY, USA). The normality of continuous variables was assessed using the Shapiro–Wilk test. As the data were not normally distributed, continuous variables are presented as median values with interquartile ranges (IQR), while categorical variables are presented as frequencies and percentages. Comparisons between the T2DM and healthy control groups were performed using the Mann–Whitney U test for continuous variables and Pearson’s chi-square test for categorical variables. The relationship between disease duration and corneal densitometry parameters was evaluated using Spearman’s rank correlation coefficient. A two-sided *p*-value < 0.05 was considered statistically significant. A power analysis was performed using G*Power version 3.1.9.7 (Heinrich Heine University Düsseldorf, Düsseldorf, Germany). For the correlation analysis, assuming a moderate correlation (ρ = 0.30), a two-sided α level of 0.05, and a target power of 80%, the required sample size was estimated at 84 patients. As the T2DM cohort comprised 45 patients, the study had limited statistical power to detect correlations of this magnitude.

## 3. Results

A total of 90 eyes were included in the analysis, comprising 45 eyes from patients with T2DM and 45 eyes from healthy controls. Both groups had an identical gender distribution, with 30 (66.7%) male and 15 (33.3%) female eyes in each group. The median age was also identical between the groups, at 59 years (IQR: 53–65 years). In the T2DM group, the median disease duration was 8 years (IQR: 4.0–15.0 years). Twenty-seven patients (60.0%) were receiving oral antidiabetic therapy, while 18 (40.0%) were treated with insulin. Diabetic retinopathy was absent in 21 (46.7%) patients, while 24 (53.3%) had non-proliferative diabetic retinopathy (NPDR). No patients with proliferative diabetic retinopathy (PDR) were included in the study. Baseline demographic and clinical characteristics are presented in Table 1.

CCT did not differ significantly between patients with T2DM and healthy controls (*p* = 0.817). Similarly, no statistically significant differences in COD were observed between the groups across any of the analyzed corneal annuli or layers. Although COD values were numerically higher in the T2DM group in several corneal regions, particularly in the 6–10 mm zone, these differences did not reach statistical significance. Detailed CCT and COD results are presented in Table 2. An exploratory subgroup analysis comparing corneal densitometry parameters between patients with T2DM with and without diabetic retinopathy is presented in Supplementary Table S1.

No statistically significant correlations were observed between disease duration and corneal densitometry measurements in patients with T2DM. Spearman’s correlation analysis showed no significant association between disease duration and COD in any of the analyzed corneal layers or annular zones (all *p* > 0.05). All correlation coefficients were weak (ρ = 0.033–0.240), and none reached statistical significance (Table 3).

## 4. Discussion

### 4.1. Principal Findings

The present study evaluated corneal densitometry in patients with T2DM using Pentacam AXL and compared the findings with those of age- and gender-matched healthy controls. No statistically significant differences in COD were observed between the groups across any of the analyzed corneal layers or annular zones. Nevertheless, median COD values were numerically higher in the T2DM group in several corneal regions, with the largest differences generally observed within the 6–10 mm annulus. CCT also did not differ significantly between the groups. Furthermore, no significant correlation was found between disease duration and COD parameters. Taken together, these findings do not provide evidence of a significant difference in corneal densitometry between patients with T2DM and matched healthy controls in the present cohort, although the numerical differences observed in some peripheral regions warrant further investigation in larger studies.

### 4.2. Corneal Densitometry in T2DM

Chronic hyperglycemia has been associated with a variety of structural and biochemical changes within the cornea, including accumulation of advanced glycation end-products (AGEs), oxidative stress, extracellular matrix remodeling, and endothelial dysfunction, all of which may potentially increase corneal light scatter and reduce transparency [5,6]. However, despite these proposed mechanisms, the present study did not demonstrate statistically significant differences in COD between patients with T2DM and age- and gender-matched healthy controls. Median COD values were numerically higher in the T2DM group in several regions, particularly within the 6–10 mm annulus, but the considerable overlap between groups and the absence of statistical significance indicate that these differences should be interpreted cautiously.

Our findings differ from several previous studies reporting significantly increased corneal densitometry in patients with diabetes. Coşkun et al. reported increased corneal densitometry across nearly all corneal layers and annular zones in patients with newly diagnosed T2DM, while Elagamy et al. observed increased corneal backscatter predominantly within the 6–10 mm annulus and total corneal layer. Özyol and Özyol similarly demonstrated higher densitometry values in diabetic patients compared with healthy controls, and Gao et al. reported increased COD accompanied by endothelial alterations in patients with diabetes [12,13,14,15]. Although the present cohort showed numerically higher COD values in several corresponding regions, particularly within the 6–10 mm annulus, these differences did not reach statistical significance.

Interestingly, the largest numerical between-group differences in the present study were observed predominantly within the 6–10 mm annulus. Previous studies have also reported more pronounced densitometric alterations outside the central corneal zone. However, given the lack of statistically significant differences in our primary analysis, the present findings cannot establish preferential involvement of the mid-peripheral cornea and should be regarded as descriptive. Further studies with larger samples are required to determine whether regional differences in susceptibility to diabetes-related corneal changes exist.

The heterogeneity of findings across studies is noteworthy. While several studies have reported increased COD in patients with diabetes, Ramm et al. reported lower COD values in diabetic eyes [17]. Differences among studies may reflect variations in study populations, age and gender distribution, disease duration, glycaemic control, severity of diabetic complications, imaging protocols, sample size, and statistical methodology. The absence of significant differences in our age- and gender-matched cohort further highlights the complexity of the relationship between diabetes and corneal densitometry and suggests that potential alterations in corneal transparency may be influenced by multiple clinical and demographic factors.

### 4.3. Central Corneal Thickness

CCT did not differ significantly between patients with T2DM and healthy controls. This observation is consistent with several previous studies that also failed to demonstrate clinically meaningful differences in CCT between patients with T2DM and healthy controls [15,20,21,22]. These findings suggest that CCT may remain relatively preserved in some patients with T2DM and may not, in isolation, adequately reflect diabetes-related corneal alterations.

Interestingly, Coşkun et al. [15] similarly reported no significant difference in CCT between age- and gender-matched patients with newly diagnosed T2DM and healthy controls, while corneal densitometry values were significantly higher across all analyzed corneal zones and depths. Based on these findings, the authors proposed that densitometric alterations may reflect subtle changes in corneal transparency before measurable alterations in corneal thickness become apparent, suggesting a potential role for corneal densitometry as a sensitive marker of early corneal involvement in T2DM [15]. In contrast, neither CCT nor COD differed significantly between groups in the present study. Differences in disease stage and cohort characteristics may partly account for these discrepant findings. Longitudinal studies incorporating both pachymetric and densitometric measurements, together with indicators of glycaemic control and disease severity, are required to clarify the temporal relationship between these corneal changes in T2DM.

### 4.4. Disease Duration and Corneal Densitometry

No statistically significant correlations were identified between disease duration and COD parameters in the present study, with all correlation coefficients being weak. Similar findings have been reported by Ramm et al., Elagamy et al., and Calvo-Maroto et al., who likewise failed to demonstrate a meaningful association between diabetes duration and COD [14,16,17].

Disease duration alone may not accurately reflect cumulative metabolic burden. Patients with a relatively short disease duration but poor glycemic control may experience greater tissue exposure to hyperglycemia than patients with longer-standing but well-controlled disease [15,23]. Unfortunately, HbA1c values were not available for analysis in the present study, preventing assessment of the relationship between long-term glycemic control and corneal transparency. Furthermore, other indicators of diabetes severity, including diabetic retinopathy and systemic complications, may contribute to variability in COD measurements. Consequently, disease duration should be interpreted alongside metabolic control and other indicators of disease severity rather than as an isolated marker of diabetes-related corneal alterations [13,20,24].

### 4.5. Clinical Interpretation

The absence of statistically significant differences in COD between patients with T2DM and matched healthy controls suggests that corneal densitometry measurements obtained with Scheimpflug imaging may not consistently distinguish patients with T2DM from healthy individuals, particularly when demographic factors are carefully balanced. Although numerically higher COD values were observed in several regions in the T2DM group, the substantial overlap between groups limits their potential clinical discriminatory value based on the present data. Corneal densitometry should therefore not currently be regarded as an established standalone biomarker of diabetic corneal involvement. Larger prospective studies incorporating measures of glycemic control and diabetes severity are needed to determine whether specific patient subgroups or corneal regions demonstrate clinically meaningful densitometric alterations.

### 4.6. Strengths and Limitations

The present study has several strengths. It provides a comprehensive regional assessment of COD by analysing both corneal depth and concentric annular zones using Pentacam AXL. Importantly, the analysis was performed in age- and gender-matched groups, reducing the potential influence of these demographic factors on between-group comparisons.

Several limitations should nevertheless be acknowledged. First, the observational cross-sectional design precludes conclusions regarding causality, temporal sequence, or longitudinal progression of corneal changes. Second, the final matched sample included 45 eyes in each group, which may have limited the ability to detect small or moderate between-group differences. This is particularly relevant given the numerical differences observed in some COD measurements despite the absence of statistical significance. Furthermore, correlation analyses with disease duration were restricted to the 45 patients with T2DM, limiting the statistical power to detect weak associations. Assuming a moderate correlation (ρ = 0.30) and a target power of 80%, the required sample size was estimated at approximately 84 patients. Consequently, the absence of statistically significant between-group differences or correlations with disease duration should not be interpreted as evidence of equivalence or absence of an association, respectively. Larger, adequately powered studies are required to determine whether smaller between-group differences or weaker associations exist.

Importantly, HbA1c values were unavailable in the present cohort. HbA1c provides an established measure of longer-term glycemic control and may more closely reflect the metabolic exposure relevant to diabetes-related tissue alterations than disease duration alone. Patients with a similar duration of T2DM may have substantially different cumulative glycemic burdens depending on the degree and persistence of hyperglycemia. Consequently, the absence of HbA1c data prevented evaluation of the relationship between glycemic control and corneal densitometry. Future studies incorporating HbA1c and other indicators of metabolic control are needed to determine whether corneal densitometric alterations vary according to chronic glycemic exposure.

Finally, although all Pentacam AXL examinations were performed by the same trained operator using the manufacturer’s quality criteria, intra-operator repeatability of corneal densitometry measurements was not specifically assessed. Consequently, study-specific repeatability metrics are not available. As corneal densitometry measurements may be influenced by image acquisition and scan quality, the absence of formal repeatability assessment should be considered when interpreting relatively small between-group differences. Future prospective studies should incorporate repeated measurements and formal repeatability analysis.

### 4.7. Future Directions

Future prospective longitudinal studies involving larger and adequately powered patient populations are needed to clarify the relationship between T2DM and corneal densitometry. Inclusion of HbA1c, diabetic retinopathy severity, systemic diabetic complications, and complementary imaging techniques such as in vivo confocal microscopy may help identify clinical factors associated with potential alterations in corneal transparency. Such studies are also needed to determine whether the small regional differences observed in the present study represent reproducible diabetes-related alterations and whether corneal densitometry has potential value in the assessment of diabetic keratopathy.

## 5. Conclusions

The study showed no statistically significant differences in corneal densitometry or central corneal thickness between patients with T2DM and healthy controls. No significant association was identified between disease duration and corneal densitometry; however, given the limited sample size, weak associations cannot be excluded, and disease duration alone may not adequately reflect diabetes-related corneal alterations. Other factors, including long-term glycemic control, disease severity, and the presence of diabetic complications, may contribute to variability in corneal densitometry. These findings highlight the complexity of diabetes-related corneal changes and suggest that corneal densitometry, when considered in isolation, may have limited ability to distinguish patients with T2DM from healthy individuals. Larger prospective longitudinal studies incorporating HbA1c, diabetic retinopathy severity, and other indicators of metabolic and disease status are warranted to determine whether subtle regional densitometric alterations occur in T2DM and to clarify the potential clinical role of corneal densitometry in diabetic keratopathy.

## Figures and Tables

**Table 1:** Baseline demographic and clinical characteristics of the study participants.

Variable	Control Group (*n* = 45)	T2DM Group (*n* = 45)
Gender [*n*, (%)]		
● Male	30 (66.7%)	30 (66.7%)
● Female	15 (33.3%)	15 (33.3%)
Age [median (IQR)]	59 (53–65)	59 (53–65)
Disease duration (years) [Median (IQR)]	NA	8 (4.0–15.0)
Therapy type [*n*, (%)]	NA	
● Oral antidiabetic drugs		27 (60.0%)
● Insulin		18 (40.0%)
Diabetic retinopathy status [*n*, (%)]	NA	
● No DR		21 (46.7%)
● NPDR		24 (53.3%)
● PDR		0 (0.0%)

IQR, interquartile range; T2DM, type 2 diabetes mellitus; DR, diabetic retinopathy; NPDR, non-proliferative diabetic retinopathy; PDR, proliferative diabetic retinopathy; NA, not applicable. Age and gender were matching variables and were therefore not subjected to between-group statistical testing. Disease duration, therapy type, and diabetic retinopathy status were applicable only to the T2DM group.

**Table 2:** Comparison of central corneal thickness and corneal optical density in control group and patients with diabetes mellitus.

Variable	Control Group (*n* = 45) Median (IQR)	T2DM Group (*n* = 45) Median (IQR)	*p*-Value
CCT (µm)	546.0 (524.5– 562.2)	544.0 (511.8– 569.2)	0.817
COD total 0–2 mm (GSU)	19.7 (19.0–20.9)	19.8 (19.2–21.0)	0.625
COD total 2–6 mm (GSU)	19.0 (18.1–20.3)	19.4 (18.7–21.4)	0.172
COD total 6–10 mm (GSU)	27.8 (23.4–33.7)	30.8 (26.9–35.6)	0.127
COD anterior 0–2 mm (GSU)	27.5 (26.3–29.5)	27.8 (26.3–30.2)	0.075
COD anterior 2–6 mm (GSU)	26.3 (24.5–27.9)	26.5 (24.9–28.8)	0.460
COD anterior 6–10 mm (GSU)	35.0 (29.5–41.7)	37.7 (33.7–44.9)	0.183
COD central 0–2 mm (GSU)	17.0 (16.3–18.1)	17.0 (16.6–18.2)	0.935
COD central 2–6 mm (GSU)	16.2 (15.7–17.6)	16.6 (16.1–18.2)	0.181
COD central 6–10 mm (GSU)	24.9 (20.6–30.2)	27.8 (24.2–32.2)	0.070
COD posterior 0–2 mm (GSU)	14.4 (13.8–15.3)	14.7 (13.9–15.6)	0.361
COD posterior 2–6 mm (GSU)	14.5 (13.6–15.6)	15.1 (13.8–16.5)	0.071
COD posterior 6–10 mm (GSU)	24.2 (20.0–27.0)	25.7 (22.4–29.0)	0.083

T2DM—type 2 diabetes mellitus, IQR—interquartile range, CCT—central corneal thickness, COD—corneal optical density, GSU—grayscale units. Between-group comparisons were performed using the Mann–Whitney U test.

**Table 3:** Correlation between disease duration and corneal densitometry parameters in diabetic eyes.

Parameter	Spearman’s Rho (ρ)	*p*-Value
COD total 0–2 mm (GSU)	0.123	0.420
COD total 2–6 mm (GSU)	0.054	0.726
COD total 6–10 mm (GSU)	0.234	0.122
COD anterior 0–2 mm (GSU)	0.161	0.291
COD anterior 2–6 mm (GSU)	0.090	0.558
COD anterior 6–10 mm (GSU)	0.240	0.112
COD central 0–2 mm (GSU)	0.080	0.603
COD central 2–6 mm (GSU)	0.033	0.829
COD central 6–10 mm (GSU)	0.192	0.207
COD posterior 0–2 mm (GSU)	0.129	0.398
COD posterior 2–6 mm (GSU)	0.039	0.801
COD posterior 6–10 mm (GSU)	0.176	0.246

COD—corneal optical density, GSU—grayscale units.

## Data Availability

The data presented in this study are available from the corresponding author upon reasonable request. The data are not publicly available because they contain information that could compromise participant privacy.

## Supplementary Materials

The following supporting information can be downloaded at: https://www.mdpi.com/article/10.3390/medicina62101887/s1, Table S1: Comparison of corneal optical density parameters between patients with T2DM without diabetic retinopathy and those with non-proliferative diabetic retinopathy.

## Abbreviations

The following abbreviations are used in this manuscript:

DM	Diabetes mellitus
T2DM	Type 2 diabetes mellitus
DR	Diabetic retinopathy
CCT	Central corneal thickness
COD	Corneal optical density
AGEs	Advanced glycation end products
GSU	Grayscale Units
BCVA	Best-corrected visual acuity
OCT	Optical Coherence Tomography
