# Supplementary material for: Evaluation of Corneal Densitometry in Patients with Type 2 Diabetes Mellitus
Source: Medicina (Kaunas). 2026 Sep 29;62(10):1887. doi: 10.3390/medicina62101887 (PMC13706833; doi:10.3390/medicina62101887)
Supplement: Supplementary file 1 [file medicina-62-01887-s001.zip › medicina-4502349-supplementary.pdf]

Supplementary Table S1. Comparison of corneal optical density parameters between patients with T2DM without diabetic retinopathy and those with non-proliferative diabetic retinopathy.

Variable	**No DR (n = 21)** Median (IQR)	**NPDR (n = 24)** Median (IQR)	**p-value**	**FDR-adjusted p-value**
COD total 0-2 mm (GSU)	19.7 (19.1-21.0)	19.8 (19.2-21.5)	0.972	0.972
COD total 2-6 mm (GSU)	19.8 (18.7-22.4)	19.3 (18.7-20.8)	0.227	0.389
COD total 6-10 mm (GSU)	31.5 (27.8-41.3)	30.3 (23.5-33.2)	0.155	0.372
COD anterior 0-2 mm (GSU)	27.4 (26.0-29.1)	27.8 (26.5-30.8)	0.327	0.491
COD anterior 2-6 mm (GSU)	26.5 (24.3-29.6)	26.5 (25.4-28.4)	0.945	0.972
COD anterior 6-10 mm (GSU)	38.2 (33.4-47.5)	37.6 (33.6-44.2)	0.608	0.730
COD central 0-2 mm (GSU)	17.2 (16.6-18.2)	16.8 (16.4-17.9)	0.368	0.491
COD central 2-6 mm (GSU)	17.5 (16.4-19.8)	16.4 (15.7-16.9)	0.015	0.068
COD central 6-10 mm (GSU)	30.3 (25.7-39.1)	27.0 (20.9-30.3)	0.065	0.195
COD posterior 0-2 mm (GSU)	15.1 (14.2-15.8)	14.5 (13.7-15.1)	0.218	0.389
COD posterior 2-6 mm (GSU)	16.4 (15.0-17.5)	14.3 (13.7-15.6)	0.003	0.036
COD posterior 6-10 mm (GSU)	26.8 (25.1-35.3)	24.5 (19.9-26.8)	0.017	0.068

DR, diabetic retinopathy; IQR, interquartile range; NPDR, non-proliferative diabetic retinopathy; CCT - central corneal thickness, COD - corneal optical density, GSU – grayscale units; FDR, false discovery rate.

P-values were obtained using the Mann–Whitney U test. The Benjamini–Hochberg procedure was applied across the 12 COD comparisons to control the false discovery rate.
